# Comparison of three reference methods for the measurement of intracellular pH using ^31^P MRS in healthy volunteers and patients with lymphoma

**DOI:** 10.1002/nbm.3047

**Published:** 2013-11-04

**Authors:** Mihaela Rata, Sharon L. Giles, Nandita M. deSouza, Martin O. Leach, Geoffrey S. Payne

**Affiliations:** ^1^ Cancer Research‐UK and EPSRC Cancer Imaging Centre at The Institute of Cancer Research and The Royal Marsden NHS Foundation TrustDivision of Radiotherapy and Imaging/MRI Unit, Downs Road, Sutton, Surrey, SM2 5PTUnited Kingdom

**Keywords:** ^31^P MRS, intracellular pH, non‐Hodgkin's lymphoma, reference peak, accuracy measurement, java‐based magnetic resonance user interface (jMRUI)

## Abstract

^31^P magnetic resonance spectroscopy (^31^P MRS) can measure intracellular pH (pH_i_) using the chemical shift difference between pH‐dependent inorganic phosphate (P_i_) and a pH‐independent reference peak. This study compared three different frequency reference peaks [phosphocreatine (PCr), α resonance of adenosine triphosphate (αATP) and water (using ^1^H MRS)] in a cohort of 10 volunteers and eight patients with non‐Hodgkin's lymphoma (NHL). Well‐resolved chemical shift imaging (CSI) spectra were acquired on a 1.5T scanner for muscle, liver and tumour. The pH was calculated for all volunteers and patients using the available methods. The consistency of the resulting pH was evaluated. The direct P_i_–PCr method was best for those spectra with a very well‐defined PCr, such as muscle (pH=7.05 ± 0.02). In liver, the P_i_–αATP method gave more consistent results (pH=7.30 ± 0.06) than the calibrated water‐based method (pH=7.27 ± 0.11). In NHL nodes, the measured pH using the P_i_–αATP method was 7.25 ± 0.12. Given that the measured range includes some biological variation in individual patients, treatment‐related changes of the order of 0.1 pH units should be detectable. © 2013 The Authors. *NMR in Biomedicine* published by John Wiley & Sons Ltd.

**Abbreviations used**
*αATP*: *α resonance of adenosine triphosphate*
*CSI*: *chemical shift imaging*
*jMRUI*: *JAVA‐based magnetic resonance user interface*
*NHL*: *non‐Hodgkin's lymphoma*
*pH_i_*: *intracellular pH*
*PCr*: *phosphocreatine*
*P_i_*: *inorganic phosphate*
*SD*: *standard deviation*

## INTRODUCTION

Most tumours, contrary to expectation, exhibit slightly alkaline values of intracellular pH (pH_i_) 1, which have been associated with a favourable environment for tumour growth 2. Inhibition of tumour growth may occur with anticancer agents that acidify the intracellular tumour environment, such as inhibitors of the monocarboxylate transporter 3 that blocks the membrane transport of the final products of glycolysis. In these instances, the measurement of drug‐induced pH_i_ changes offers the opportunity to verify the mechanism of drug action.

Measurement of pH_i_ using ^31^P MRS was first proposed about 30 years ago 4. The initial human applications included muscle exercise studies 5, 6 and the detection of some muscle pathologies, such as the glycogen storage disease McArdle's syndrome 7. The measurement of pH by ^31^P MRS 4, 8 relies on the chemical shift difference between pH‐dependent inorganic phosphate (P_i_) and a pH‐independent reference peak. *In vivo* ^31^P MRS has the advantage of being a noninvasive and nonperturbing technique, enabling longitudinal clinical studies to be performed through a course of treatment.

Three internal frequency references have been suggested to measure pH_i_: two ^31^P‐derived peaks and a ^1^H‐derived peak. The phosphocreatine (PCr) peak is widely used for pH measurement in muscle 9, 10, 11 as a result of its strong nonoverlapping single resonance, but, in other tissues, as well as in tumours, it may be less suitable owing to its low concentration. One alternative is to use the α resonance of adenosine triphosphate (αATP) 12, 13, as this compound is visible in most spectra and is relatively insensitive to pH and ion content 4. A third method is based on the ^1^H MRS signal from tissue water acquired from the same region 14, whose resonance frequency is scaled to create a ‘virtual reference’ peak in the ^31^P spectrum. Although this method requires a dual‐receiver coil, the water resonance is a well‐defined single peak with a very good signal‐to‐noise ratio. In addition, technology has improved since the initial evaluation of the method, permitting simplification of the measurement protocol, particularly with regard to digital resolution of the frequency synthesiser, while modern shielded gradients substantially reduce the field shift from eddy currents.

These methods have all been available for some time. However, given the recent interest in exploring the use of pH_i_ as a biomarker of some new targeted anticancer therapies, it is timely to compare the three methods on modern hardware, and to obtain estimates of their precision. In this study, we calibrated the water reference method in human calf (gastrocnemius) muscle, compared with PCr, αATP and the water reference in muscle and liver (in volunteers), and applied the best method to obtain a measurement of pH in a pilot cohort of patients with non‐Hodgkin's lymphoma (NHL).

Several other ^31^P MRS methods of measuring pH based on chemical shifts among the ATP peaks 15 or between βATP and PCr peaks 16 are available. In addition to pH value measurement, some of these methods can simultaneously estimate intracellular [Mg^2+^] concentration. Such complex equations, however, require further calibrations to determine unknown constants for each study design. In the present study, we focused on comparing the most established pH‐dependent peak (P_i_) with three options for the reference peak.

## EXPERIMENTAL

### Volunteers and patients

Data were obtained from 10 healthy volunteers, aged 24–58 years (five right gastrocnemius muscle and five liver spectra), and eight newly diagnosed patients with NHL, aged 38–79 years (three abdomen, three chest, one neck and one knee spectra). The NHL subtypes were; four diffuse large B‐cell lymphomas, two follicular lymphomas and two mixed lymphomas (diffuse large B‐cell lymphoma + follicular lymphoma). All volunteers and patients gave written informed consent and the scanning protocol was approved by the local ethical committee. Only patients with superficial and/or bulky tumour mass (>2 × 2 × 2 cm^3^ on prior computed tomography scan) were included in the study.

### MR examination

All data were acquired using a 1.5 T Avanto scanner (Siemens, Erlangen, Germany) using three custom‐built dual ^1^H/^31^P surface coils 17, 18, 19 of 5, 8 and 12 cm in diameter. In the volunteer measurements, the 8 cm coil was chosen for the superficial calf muscle examination, whereas the 12 cm coil offered better coverage of the whole liver. For each patient, the appropriate surface coil was chosen individually, based on tumour size and location. Attention was paid to position the acquisition voxel within viable tumour tissue, excluding large blood vessels, necrotic or cystic regions.

The MR protocol consisted of: a) three orthogonal anatomical images for planning the spectral acquisition; b) ^1^H single voxel spectroscopy; and c) 3D ^1^H‐decoupled ^31^P chemical shift imaging (CSI). The anatomical images were based on a *T*_2_ steady‐state gradient echo sequence with the following parameters: field of view, 400 mm; TR/TE = 3.79/1.9 ms; 12 slices with a thickness of 7 mm. A water spectrum was acquired using a ^1^H MRS point‐resolved spectroscopy sequence (with TR/TE = 2000/135 ms; vector size, 1024; bandwidth, 1000 Hz; 60 averages; total acquisition time, ~2 min). The transmit frequency of the ^1^H MRS acquisition was centred to the water frequency following the standard procedure of the scanner software. The ^31^P CSI protocol (TR = 1000 ms; RF pulse calibrated to 45° at the voxel centre; vector size, 1024; 8 × 8 × 8 phase‐encoding steps; two averages; spectral width, 2000 Hz; full k‐space coverage with 100% Hamming filter; total acquisition time, ~17 min) acquired information from the same voxel as for the water spectrum. The Hamming filter was applied in scanner software to smooth the truncation effect caused by the limited coverage of *k* space (eight steps). The ^31^P transmit frequency was set in two steps. Firstly, the frequency was calibrated using a ^31^P reference sample (triphenyl phosphate) sited within the ^31^P coil housing. Secondly, the scanner frequency was offset from this reference frequency by a known value (–3563 Hz) to centre the spectrum relative to the metabolites (centre frequency between γATP and αATP). All volunteer spectra (muscle or liver) were derived from a 27 mL isotropic voxel. The patient voxel size varied from 15.6 to 125 mL depending on the tumour volume. The same shimming was used for both ^1^H and ^31^P acquisitions. No water spectra were acquired in the patient cohort.

To test for frequency shifts from eddy current effects, localised and unlocalised phantom data were acquired for both ^1^H and ^31^P MRS using the 5 cm ^1^H/^31^P coil. Two spherical phantoms of 3 cm in diameter were used. The ^1^H phantom contained a 0.13 mM MnCl_2_ water solution, and the ^31^P phantom contained 0.1 M NaH_2_PO_4_ doped with 0.24 mM NiCl_2_. Both phantoms were positioned slightly off‐isocentre (~10 cm laterally) similar to most *in vivo* locations. As phantom data were expected to have a good signal‐to‐noise ratio and not to suffer from any motion, the number of averages used for *in vivo* acquisitions could be reduced. The phantom data were hence acquired with 10 averages for the ^1^H single‐voxel spectroscopy and one average for ^31^P CSI.

### Post‐processing and pH measurement

Spectra were processed using the JAVA‐based magnetic resonance user interface (jMRUI) v.5 software and quantified using a nonlinear least‐squares algorithm [AMARES 20]. pH values were calculated using three reference peak methods for the volunteer data and two for the patient data. The pH calculation used the following calibrated form of the Henderson–Hasselbalch equation 11, 13:

$$\mathrm{pH}=6.75+\log_{10}\left[\left(\delta –3.27\right)/\left(5.69–\delta \right)\right]$$

where *δ* is the chemical shift frequency difference between pH‐dependent P_i_ and a pH‐independent reference peak, measured in parts per million (ppm).

Method 1 (PCr based) used the chemical shift difference *δ* between P_i_ and PCr:

$$\delta =f_{\mathrm{Pi}}-f_{\mathrm{PCr}}$$

Method 2 (αATP based) used the same equation, but with *δ* calculated from the chemical shift of αATP:

$$\delta =f_{\mathrm{Pi}}-f_{\mathrm{\alpha ATP}}-7.56$$

where 7.56 ppm represents the expected frequency of the centre of the αATP doublet peak relative to PCr.

**Figure 1:**
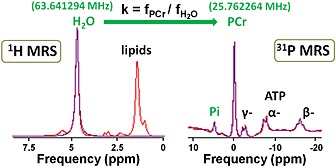
Example of ^1^H and ^31^P MR spectra acquired at 1.5 T in a healthy volunteer muscle used to calibrate the water‐based method. H_2_O, water; PCr, phosphocreatine; P_i_, inorganic phosphate; α, β, γ, nucleoside triphosphates. Peaks of interest of each spectrum are labelled in green. For this particular example, the measured frequencies were $f_{H_{2}O}$ = 63.641294 MHz and *f*_PCr_ = 25.762264 MHz, giving *k* = 0.40480421. The frequency of water signal from the ^1^H spectrum was multiplied by the experimentally derived constant *k* to create a virtual peak in the ^31^P spectrum, called the estimated PCr. Subsequently, pH was calculated using the Henderson–Hasselbalch equation and the chemical shift difference between the measured P_i_ and the estimated PCr.

Method 3 [water‐derived PCr reference, a simplified method of Madden *et al*. 14] used the water signal from the ^1^H spectrum, acquired from the same voxel as for ^31^P MRS, to estimate the frequency of the PCr peak in the ^31^P spectrum (see Fig. 1):

$$f_{\mathrm{PCr}}=f_{H_{2}O}\cdot k$$

This method relies on the fact that the absolute frequency positions (in Hz) of both water and PCr are proportional to the local magnetic field *B*_local_, which is the main magnetic field *B*_0_ affected by any local susceptibility effects. The absolute frequency of PCr is then defined as:

$$f_{\mathrm{PCr}}=\frac{\gamma_{31_{P}}\cdot B_{\mathrm{local}}\cdot \left(1-\sigma_{\mathrm{PCr}\;}\right)}{2\pi }$$

where $\gamma_{31_{P}\;}$is the ^31^P gyromagnetic ratio and *σ*_PCr_ is the chemical shielding effect for PCr. The absolute frequency of water is similarly defined and, by dividing the two equations, we obtain:

$$\frac{f_{\mathrm{PCr}}}{f_{H_{2}O}}=\frac{\frac{\gamma_{31_{P}}\cdot B_{\mathrm{local}}\cdot \left(1-\sigma_{\mathrm{PCr}}\right)}{2\pi }}{\frac{\gamma_{1_{H}}\cdot B_{\mathrm{local}}\cdot \left(1-\sigma_{H_{2}O}\right)}{2\pi }}=\frac{\gamma_{31_{P}}\cdot \left(1-\sigma_{\mathrm{PCr}}\right)}{\gamma_{1_{H}}\cdot \left(1-\sigma_{H_{2}O}\right)}=k=\mathrm{constant}.$$

Thus, once one has measured or calculated *k*, one can use the previous relation to estimate the PCr frequency from the water frequency. In the previous work of Madden *et al*. 14, it was necessary to include corrections for limited frequency resolution of the synthesiser, and for frequency shifts caused by eddy currents. These should not be required with modern hardware, but localised pulse‐acquire spectra were acquired in a test object to check that there was no frequency shift from eddy current effects.

The multiplication factor *k* was calibrated experimentally in *in vivo* muscle datasets exhibiting high PCr, and then applied in liver spectra. The same equation as for Method 1 was subsequently applied.

## RESULTS AND DISCUSSION

^1^H phantom and ^31^P phantom data were acquired in both localised and unlocalised spectra. The ^1^H MRS measured a water peak at the same frequency (0 Hz) for both types of acquisition. Similarly, a ^31^P signal was acquired at the same position (131.83 Hz) for localised *versus* unlocalised spectra. The sampling interval was 1 Hz for all ^1^H and ^31^P experiments. These results suggest that, if present, effects from eddy currents were smaller than 1 Hz. Therefore, no eddy current corrections were applied to further acquisitions.

**Figure 2:**
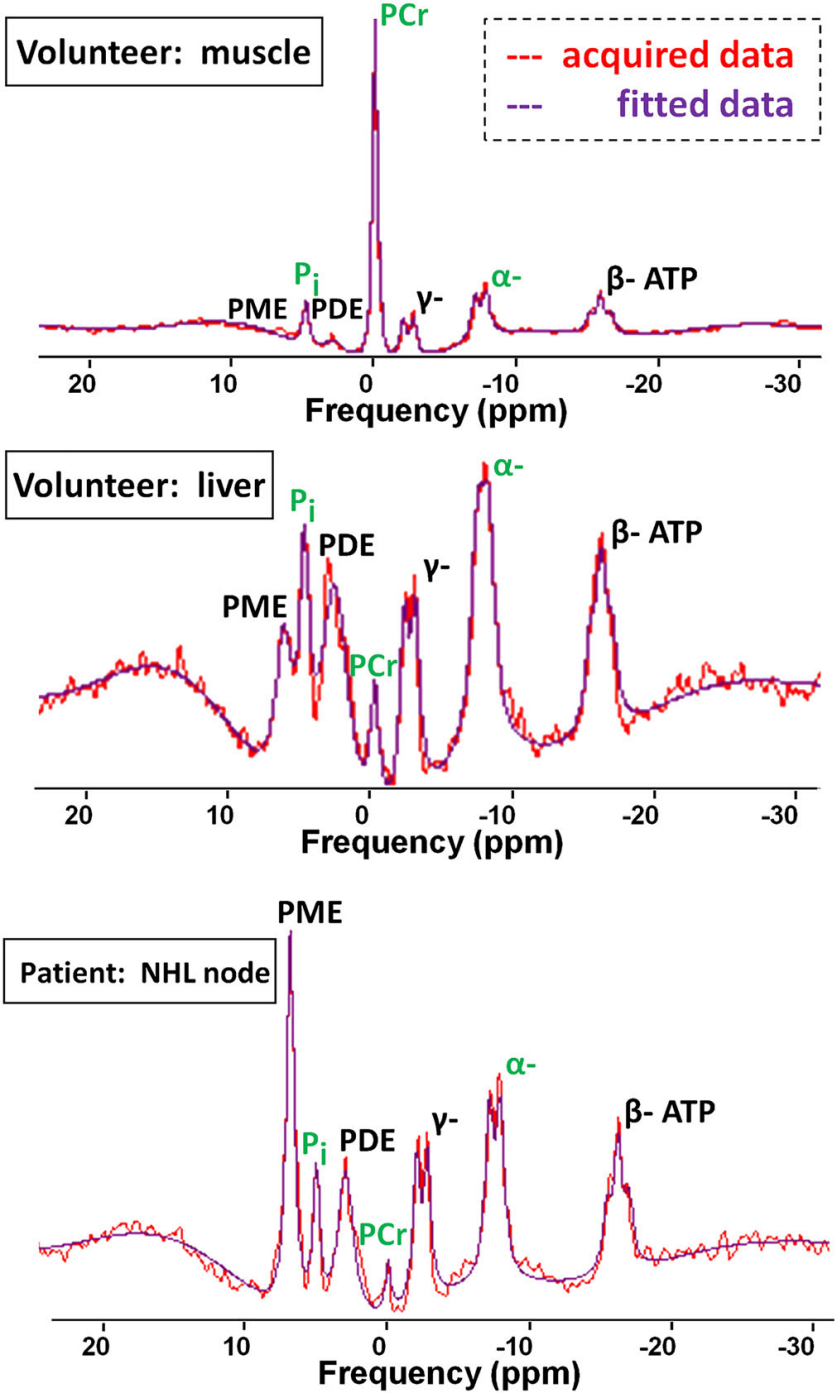
Example ^31^P MR spectra acquired at 1.5 T for muscle, liver and non‐Hodgkin's lymphoma (NHL). PCr, phosphocreatine; PDE, phosphodiesters; P_i_, inorganic phosphate; PME, phosphomonoesters; α, β, γ, nucleoside triphosphates. Peaks of interest are labelled in green.

Well‐resolved spectra were acquired from healthy volunteers and patients with NHL despite the relatively deep position of some voxels (depth range, 4–8 cm from the coil). Figure 2 illustrates example fitted spectra for each type of data acquisition. Small, but measurable, PCr peaks were observed in liver and tumour spectra. The lack of motion gating, however, meant that contamination from intense PCr signals of superficial muscle may have contributed to these peaks.

In muscle, the average value of the constant *k* between the ^31^P MRS frequency of PCr and the ^1^H MRS frequency of water was 0.404804239 ± 0.000000015. The mean measured position of the water reference in the ^31^P spectra was 0.0004 ± 0.0367 ppm.

pH values in the three tissues using the different methods are shown in Table 1. pH measurements in muscle were the most consistent, probably owing to the well‐defined PCr peak and absence of tissue motion, with Method 1 (P_i_–PCr) exhibiting a minimal standard deviation (SD) (0.02 pH units). In liver, the PCr peak was less clearly detected and may have arisen from muscle outside the selected voxel; the αATP and water reference methods were most consistent, with the P_i_–αATP method giving the smallest variation of 0.06 pH units.

**Table 1:** Intracellular pH measurements (mean ± SD) in healthy volunteers and patients with non‐Hodgkin's lymphoma (NHL) using three different methods

	PCr based		αATP based		Water based	
pH measurement	Mean	SD	Mean	SD	Mean	SD
Volunteer: muscle	7.05	0.02	7.00	0.03	7.03	0.04
Volunteer: liver	7.21	0.12	7.30	0.06	7.27	0.11
Patient: NHL	7.17	0.17	7.25	0.12	na	na

na, not applicable.

This study analysed data from volunteers and patients in a sequential manner. Observations derived from the analysis of volunteer data generated slight modification of the acquisition protocol for the patient cohort. All three methods of measurement of pH presented here were tested on volunteers (muscle and liver tissue), but only two were further applied to tumours in patients. The water‐derived method failed to demonstrate improvement in accuracy of measurement for both muscle and liver data. Therefore, in order to avoid extra acquisition with no expected benefit, no water acquisition was performed for the patient cohort.

In tumour (NHL), the P_i_–αATP method yielded a slightly alkaline pH of 7.25 with a much smaller SD (0.12) than using the PCr method (0.17). Among the three types of tissue investigated here, the greatest variation in the measurement of pH was in tumours, but, even here, the variation was relatively low (0.12 pH units). Given the biological variation of tumours, these results are encouraging and should result in a good intra‐patient repeatability.

The performance of the water reference method in liver was disappointing, given the good signal‐to‐noise ratio and well‐defined single resonance of the water peak. Although the use of two separate measurements tends to increase uncertainty, the consistent result in muscle (SD = 0.04 ppm) shows that intrinsically the method is working well. Factors such as digital resolution, amplifier frequency linearity between ^31^P and ^1^H, and arithmetic precision of the analysis were the same for muscle and liver measurements. However, in liver, the slightly larger water linewidths (12.6 Hz in liver, compared with 10.7 Hz in muscle) and the presence of motion may have affected the result.

The conclusion of this study used direct comparison of the SD values for each method and tissue. A statistical analysis of variance, such as *F*‐test (two pH methods) and Bartlett test (three pH methods), was performed. The results may be regarded with caution because of the small sample size (five and eight data points). The reported *p* values (at a significance level of 95%) were 0.31 (muscle), 0.5 (liver) and 0.35 (tumour). These statistical tests show no significant difference between the measured SD values of the different methods. However, although the statistical analysis does not support a preferred method, two further considerations influence a recommendation. The water method (with its extended acquisition time) showed no benefit for the quality of pH measurement. The PCr–P_i_ method could result in the PCr peak visible in liver/tumour spectra being contaminated from adjacent muscle tissue, making this method less reliable. For these reasons, we recommend the use of the αATP method for liver and tumour acquisitions.

It is generally acknowledged that the P_i_ peak has both intra‐ and extracellular components. In this study, only one single P_i_ peak of the MR spectrum was resolved, implying that the measured pH was a weighted average of intracellular and extracellular pH. However, the contributions from pH_i_ are expected to be at least 85% of the total pH 1. Such a contribution of the intracellular volume to the measured pH can be estimated when the total tumour volume and the fractional volume of extracellular water are known 21. Calculations show that, if the extracellular volume does not exceed 55%, then pH measured by ^31^P MRS largely represents pH_i_ 22. This extracellular compartment is expected to be larger in tumour tissue than in normal tissue owing to necrotic or cystic regions. The extracellular contamination was minimised here by careful positioning of the voxel of interest to avoid such regions.

Overall repeatability, will be influenced by a combination of factors, e.g. voxel repositioning uncertainty, coil sensitivity, shimming quality, subject motion and random noise. To detect drug response in the context of measurement variability, repeatability acquisitions are desirable in any longitudinal trial. The noninvasive nature of the examination and the lack of need for an extrinsic contrast agent make this easier to implement. The ^31^P MRS method (including images and calibrations), however, requires a total scanning time of about 40 min per visit and its application is limited to superficial tumours of about 2–3 cm in size, or to larger tumours if deeper, for signal detection using a surface coil.

In conclusion, pH was successfully calculated for all volunteers and patients. The direct P_i_–PCr method is best only for spectra with very well‐defined PCr, such as muscle. The P_i_–αATP method measured pH in liver with an SD value of 0.06 units, and in tumours with an SD value of 0.12 pH units, and is preferred in these tissues.
